# Geographic remoteness and risk of advanced colorectal cancer at diagnosis in Queensland: a multilevel study

**DOI:** 10.1038/bjc.2011.356

**Published:** 2011-09-06

**Authors:** P D Baade, P Dasgupta, J Aitken, G Turrell

**Affiliations:** 1 Viertel Centre for Research in Cancer Control, Cancer Council Queensland, Spring Hill, Brisbane, QLD 4004, Australia; 2 School of Public Health, Queensland University of Technology, Brisbane, QLD 4059, Australia; 3 Griffith Health Institute, Griffith University, Gold Coast, QLD 4222, Australia; 4 School of Population Health, University of Queensland, Brisbane, QLD 4061, Australia

**Keywords:** colorectal cancer, stage, health inequality, rural health, socio-economic status

## Abstract

**Background:**

We examine the relationships between geographic remoteness, area disadvantage and risk of advanced colorectal cancer.

**Methods:**

Multilevel models were used to assess the area- and individual-level contributions to the risk of advanced disease among people aged 20–79 years diagnosed with colorectal cancer in Queensland, Australia between 1997 and 2007 (*n*=18 561).

**Results:**

Multilevel analysis showed that colorectal cancer patients living in inner regional (OR=1.09, 1.01–1.19) and outer regional (OR=1.11, 1.01–1.22) areas were significantly more likely to be diagnosed with advanced cancer than those in major cities (*P*=0.045) after adjusting for individual-level variables. The best-fitting final model did not include area disadvantage. Stratified analysis suggested this remoteness effect was limited to people diagnosed with colon cancer (*P*=0.048) and not significant for rectal cancer patients (*P*=0.873).

**Conclusion:**

Given the relationship between stage and survival outcomes, it is imperative that the reasons for these rurality inequities in advanced disease be identified and addressed.

While colorectal (CRC) survival rates in Australia are among the highest in the world (Coleman *et al*, 2011), people living outside major cities or in disadvantaged areas have poorer prognosis (Yu *et al*, 2005; Kelsall *et al*, 2009). Since stage at diagnosis is a major predictor of long-term CRC outcomes (Altekruse *et al*, 2010), its relationship to factors such as socio-economic status (SES) and geographic remoteness is of particular relevance for cancer control. With major medical centres being concentrated in densely populated urban centres, it has been suggested that rural and remote locations may be associated with poorer access to screening and specialised health care (Parikh-Patel *et al*, 2006; Heathcote and Armstrong, 2007). However ecological studies have presented a mixed picture in terms of SES (Parikh-Patel *et al*, 2006; Frederiksen *et al*, 2008; Henry *et al*, 2009; Booth *et al*, 2010) and geographic remoteness (Fazio *et al*, 2005; McLafferty and Wang, 2009; Sankaranarayanan *et al*, 2009) in stage at diagnosis.

Most of the evidence is based on ecological studies and these are not able to separate the area-level or individual-level influences (Baade *et al*, 2010), limiting our understanding about area-level health inequalities. To date, no Australian study has employed multilevel methods to investigate links between geographic remoteness, area disadvantage, individual-level factors and advanced CRC.

## Materials and methods

Ethical approval to conduct this study was obtained from the University of Queensland and Queensland Health. Data for individuals aged 20–79 years diagnosed with invasive stage 1–IV CRC (ICD-O3 codes C18 to C20, C21.8) in Queensland between 1 January 1996 and 31 December 2007 (inclusive) (*n*=18 561) with complete address information were extracted from the Queensland Cancer Registry (QCR).

Information extracted from pathology forms (Krnjacki *et al*, 2008) was used to determine stage at diagnosis according to TNM system (Sobin and Wittlekind, 2002) as described previously (Baade *et al*, 2011). For multivariate analysis, localised cases (Stages I–II) were considered as early stage (Parikh-Patel *et al*, 2006; Henry *et al*, 2009) while regional and distant cases were categorised as ‘advanced’ based on their lower survival rates (Altekruse *et al*, 2010).

Information was obtained from QCR on individual-level variables: year and age of diagnosis, cancer site, gender, marital status, occupation (Turrell *et al*, 2007) and indigenous status (see Table 2 for categories).

Statistical Local Areas (SLAs), which are typically based on local governments and councils and thus likely to be socio-economically relevant to their residents, were used as the geographical definition for area-level analysis (Baade *et al*, 2010). Remoteness of residence was defined using the Accessibility/Remoteness Index of Australia (ARIA+) classification (AIHW, 2004) and area-level socio-economic disadvantage measured using the Index of Relative Socio-economic Disadvantage (IRSD) (Australian Bureau of Statistics, 2006) which categorises SLAs into five quintiles of increasing advantage from quintile 1.

### Modelling

Multilevel logistic modelling (MLwiN 2.21) using Markov Chain Monte Carlo (Browne, 2009) approaches in MLwiN version 2.21 (University of Bristol, UK) was used. Chain convergence was checked using Raftery-Lewis diagnostics. Models were compared using the Bayesian deviance information criterion (DIC; Spiegelhalter *et al*, 2002), with smaller values indicating better fit.

Analyses were conducted in three steps: (1) a null model comprising individuals (Level 1) nested in SLAs (Level 2) with no fixed effects; (2) extending to include individual-level factors as fixed effects (Model 2); and (3) geographic remoteness (Model 3) and neighbourhood disadvantage (Model 4) were included separately as fixed effects to quantify how much area variation in stage was due to these factors independent of compositional effects, and then in combination (Model 5). Fixed effects results are reported as odds ratios (95% CI) (Merlo *et al*, 2001; Eikemo *et al*, 2008). Significance of individual coefficients was tested using *Z* test.

## Results

Overall, 57.1% of patients were male with 67.2% having colon cancer. The mean age at diagnosis was 65 years (median=66 years). About 44.8% were diagnosed with advanced CRC. Just over half of the patients (57.6%) lived in major cities and around 36.5% were in the two most affluent SES quintiles.

In the multivariable logistic regression analyses, the null model (Model 1) indicated significant (*P*=0.041) between area variations across the SLAs (Table 1).

Based on the DIC statistic (Table 1), the model fit improved substantially by including individual-level characteristics (Model 2) and then geographic remoteness (Model 3). Adding area disadvantage (Model 4) to Model 2 did not improve the fit. Similarly, the full model (Model 5) provided a poor fit to the data than Model 3, suggesting that Model 3 was the best-fitting model for these data. There was no evidence for area-level interaction (results not shown).

In this final model (Model 3), and independent of individual factors, geographic remoteness was associated with cancer stage (Table 2). At individual level sex, occupation, indigenous status and anatomic site were independent predictors (*P*<0.001) of advanced CRC (Table 2). Independent of area effects the likelihood of advanced CRC was significantly higher for females than for males; blue-collar workers *vs* professionals; individuals with known indigenous status compared with unknown and patients with colon rather than rectal cancer (Table 2).

Analyses stratified by cancer site (Model 3) (results not shown) showed that area remoteness was significant for colon cancer (*P*=0.048) but not for rectal cancer (*P*=0.873).

## Discussion

This study is one of the first to consider geographical variations in CRC stage at diagnosis after adjusting for both area- and individual-level factors. We found significant evidence that a person's risk of being diagnosed with advanced CRC depends on where they live, specifically for those diagnosed with colon cancer, independently of the individual characteristics of the patient themselves. The impact of geographical location, however, was limited to rurality with no evidence that area disadvantage was associated with stage at diagnosis.

Given the nature of our data, any discussion of the possible reasons for the remoteness differential can only be speculative; but these may include a relative shortage of experienced medical staff in regional areas and greater difficulty of accessing diagnostic services.

Significantly higher risks of late-stage diagnosis were seen for patients with colon *vs* rectal cancer, consistent with international studies (Frederiksen *et al*, 2008; Sankaranarayanan *et al*, 2009). We also found that the risk of advanced disease was higher in more regional areas compared with major cities for colon cancers only. A contributing reason for both these observations may be that compared with colon cancer rectal cancer often presents with more visible symptoms (Majumdar *et al*, 1999), thereby making patients more likely to seek medical care and be diagnosed earlier.

The strengths of this study include the use of staged CRC cases from a large, unselected, state-wide population-based registry. Approximately 84% of records in our initial cohort had sufficient information to be staged similar to that reported elsewhere (Yu *et al*, 2008). We were limited to the individual-level SES variable of occupation, since the QCR does not collect information about education (Frederiksen *et al*, 2008), income (Frederiksen *et al*, 2008) or private insurance status (Halpern *et al*, 2009) known to be associated with advanced CRC. In addition, due to the high prevalence of advanced CRC the odds ratios may reflect an overestimation of the relative risk.

## Conclusion

Given the relationship between stage at diagnosis and survival outcomes, it is imperative that the reasons for the geographical inequities in advanced disease be identified and addressed.

## Figures and Tables

**Table 1:** Random effects

	**Model 1**		**Model 2**		**Model 3**		**Model 4**		**Model 5**	
Area variance and standard error	0.013	0.007	0.010	0.006	0.009	0.006	0.009	0.006	0.009	0.006
*P*-value for area variance	0.041		0.091		0.108		0.107		0.118	
Percentage reduction in area variance from the null model	—		23.1		30.8		30.8		30.8	
DIC	25 526.1		24 901.1		24 896.2		24 905.8		24 902.5	

Abbreviation: DIC=Deviance Information Criterion.

Model 1: no fixed effects.

Model 2: adjusted for sex, age, year, marital and indigenous status, occupation and cancer site.

Model 3: adjusted for sex, age, year, marital and indigenous status, occupation, cancer site and remoteness.

Model 4: adjusted for sex, age, year, marital and indigenous status, occupation, cancer site and area disadvantage.

Model 5: adjusted for sex, age, year, marital and indigenous status, occupation, cancer site, remoteness and area disadvantage.

**Table 2:** Final fixed effect factors on the probability of experiencing advanced stage colorectal cancer, Queensland, 1996–2007

	**Model 3**	
**Fixed effects**	**OR**	**95% CI**
*Area-remoteness index of Australia*		
Major city	1.00	—
Inner regional	1.09	1.01, 1.19
Outer regional	1.11	1.01, 1.22
Remote/very remote	1.00	0.85, 1.16
*P*-value	0.045	
		
*Year of diagnosis*		
1996–1998	1.00	
1999–2001	0.92	0.85, 1.01
2002–2004	0.99	0.91, 1.08
2005–2007	1.09	1.00, 1.19
*P*-value	<0.001	
		
*Age (years)*		
20-49	1.12	1.00, 1.26
50-59	1.10	1.01, 1.20
60-69	1.07	1.00, 1.15
70-79	1.00	
*P*-value	0.072	
		
*Sex*		
Male	1.00	—
Female	1.21	1.13, 1.29
*P*-value	<0.001	
		
I*ndigenous status*		
Non-indigenous	1.00	—
Indigenous	0.96	0.70, 1.32
Not stated	0.49	0.43, 0.56
*P*-value	<0.001	
		
*Occupation*		
Professional	1.00	—
White collar	1.00	0.90, 1.11
Blue collar	1.09	0.99, 1.20
Not in the labour force	0.62	0.57, 0.69
Not stated	0.46	0.41, 0.51
*P*-value	<0.001	
		
*Marital status*		
Married	1.00	
Never married	1.04	0.92, 1.17
Widowed	0.89	0.81, 0.98
Divorced	1.04	0.93, 1.15
Separated	0.89	0.73, 1.10
Unknown	0.97	0.75, 1.27
*P*-value	0.153	
		
*Cancer type*		
Rectal	1.00	—
Colon	1.20	1.13, 1.28
*P*-value	<0.001	

Abbreviations: OR=odds ratio; CI=confidence interval.
